# Supplementary material for: Orally Induced Hyperthyroidism Regulates Hypothalamic AMP-Activated Protein Kinase
Source: Nutrients. 2021 Nov 24;13(12):4204. doi: 10.3390/nu13124204 (PMC8708331; doi:10.3390/nu13124204)
Supplement: Supplementary file 1 [file nutrients-13-04204-s001.zip › nutrients-1431400-supplementary.pdf]

Supplementary Table S1. RT-PCR primers and probes

mRNA	GenBank		Sequence
UCP1	NM_009463.3	Assay ID	ThermoFisher TaqMan® Gene Expression Assays<br>Assay ID Mm01244861_m1
CIDEA	NM_007702.2	Assay ID	ThermoFisher TaqMan® Gene Expression Assays<br>Assay ID Mm00432554_m1
Pparg	NM_011146.3	Assay ID	ThermoFisher TaqMan® Gene Expression Assays<br>Assay ID Mm01184322_m1
Ppargc1a	NM_008904.2<br>NR_027710.1	Assay ID	ThermoFisher TaqMan® Gene Expression Assays<br>Assay ID Mm01208835_m1
Prdm16	NM_001177995.1<br>NM_001291026.1<br>NM_001291029.1<br>NM_027504.3	Assay ID	ThermoFisher TaqMan® Gene Expression Assays<br>Assay ID Mm01266512_m1
Dio2	NM_010050.2	Assay ID	ThermoFisher TaqMan® Gene Expression Assays<br>Assay ID Mm00515664_m1
Dio3	NM_172119.2	Assay ID	ThermoFisher TaqMan® Gene Expression Assays<br>Assay ID Mm0051548953_s1
Hr	NM_021877.3	Assay ID	ThermoFisher TaqMan® Gene Expression Assays<br>Assay ID Mm00498963_m1
Klf9	NM_010638.4	Assay ID	ThermoFisher TaqMan® Gene Expression Assays<br>Assay ID Mm00495172_m1
Aldh1a1	NM_013467.3	Assay ID	ThermoFisher TaqMan® Gene Expression Assays<br>Assay ID Mm00657317_m1
TRα	NM_178060.3	Assay ID	ThermoFisher TaqMan® Gene Expression Assays<br>Assay ID Mm00617505_m1
TRβ	NM_001113417.1<br>NM_009380.3	Assay ID	ThermoFisher TaqMan® Gene Expression Assays<br>Assay ID Mm00437044_m1
Hprt	NM_012583	Sense<br>Antisense<br>Probe	5’-AGCCGACCGGTTCTGTCAT-3'<br>5’-GGTCATAACCTGGTTCATCATCAC-3'<br>FAM-5’-CGACCCTCAGTCCCAGCGTCGTGAT-3’-TAMRA
